# Effects of Replacing Cow’s Milk with Plant-Based Beverages on Potential Nutrient Intake in Sustainable Healthy Dietary Patterns: A Case Study

**DOI:** 10.3390/nu16183083

**Published:** 2024-09-13

**Authors:** Paola Biscotti, Massimiliano Tucci, Donato Angelino, Valentina Vinelli, Nicoletta Pellegrini, Cristian Del Bo’, Patrizia Riso, Daniela Martini

**Affiliations:** 1 Department of Food, Environmental and Nutritional Sciences (DeFENS), Università Degli Studi di Milano, 20133 Milan, Italy; 2 Department of Bioscience and Technology for Food, Agriculture and Environment, University of Teramo, 64100 Teramo, Italy; 3 Department of Agricultural, Food, Environmental and Animal Sciences, University of Udine, 33100 Udine, Italy

**Keywords:** cow’s milk, plant-based drinks, milk alternatives, dietary patterns, nutritional adequacy, sustainability

## Abstract

More consumers are replacing cow’s milk (CM) with plant-based drinks (PBD), but data indicating the nutritional impact are limited. This theoretical study aims to assess the effect of substituting CM with PBD sold in Italy on nutrient intake within two dietary patterns: one aligned with the EAT-Lancet Commission reference diet adapted to Italian food habits (EAT-IT) and another one in line with the Italian Dietary Guidelines (IDG). Nutrition information from 368 PBD were collected and categorized according to their descriptive name and their fortification or not with calcium (Ca- and nCa-fortified). The substitution of CM with each PBD category in both dietary patterns was conducted, and an analysis of nutrient content and adequacy was performed. Substituting CM with all PBD resulted in reduced protein intake, except for nCa-fortified soy drinks, decreased saturated fat and vitamins B2 and B12, and increased fiber intake. Replacing CM with nCa-fortified PBD within both diets decreased Ca intake. Following the substitution of CM with Ca-fortified PBD, variations in vitamin D intake depended on the PBD category. The main risk of nutritional inadequacy was observed in Ca and vitamin D levels, which may even be amplified considering the different bioavailability based on the source of nutrients. This study highlighted the important role of CM in meeting calcium requirements and the potential unintended consequences of substituting CM with PBD without considering their nutritional differences.

## 1. Introduction

A large body of evidence demonstrates that suboptimal diets are responsible for more deaths than any other risk for the development of non-communicable diseases [1]. At the same time, there is an increasing awareness of the large impact of the food systems on the environment [1,2]. In addition, the world population is increasing, and it is expected to reach 9.8 billion in 2050 [3]. For all these reasons, a critical challenge faced by global food systems is to be able to provide sufficient foods—in terms of both quality and quantity—to an increasing population without depleting the earth’s resources and crossing planetary boundaries [2]. Considering the significant impact of suboptimal diets on health and the environment, the concept of sustainable diets, defined by the Food and Agriculture Organization of the United Nations (FAO) in 2010 and updated in 2019, is gaining increasing attention [4]. Indeed, the FAO and the World Health Organization (WHO) defined sustainable healthy diets as “dietary patterns that promote all dimensions of individuals’ health and wellbeing; have low environmental pressure and impact; are accessible, affordable, safe and equitable; and are culturally acceptable [5,6]. 

In 2019, the EAT-Lancet Commission proposed the first global reference diet (i.e., the EAT-Lancet healthy reference diet—ELHRD, or planetary health diet—PHD), promoting both human and planetary health, characterized by a high potential of local adaptation and scalability [7]. Subsequent studies investigated how to adapt the ELHRD to specific cultures, contexts, income levels, and geographical settings [8,9,10,11,12]. As an example, Tucci and colleagues developed a Mediterranean dietary pattern aligned with the ELHRD and adapted to Italian food habits (EAT-IT) [11,13]. The ELHRD is characterized by a high intake of whole grains, vegetables, fruits, legumes, and nuts, while limiting the consumption of animal-based foods, added sugar, and saturated fat (SFA) [7,14]. Recognizing this shift as fundamental to achieving healthy and sustainable diets, the EAT-Lancet Commission provided guidance for an increased consumption of plant-based foods and a reduction in animal food intake [7]. Indeed, a large body of evidence demonstrated that shifting towards plant-based diets would promote human health and reduce the environmental impact of diet-related activities, such as land use, greenhouse gas emissions, eutrophication, green and blue water use [15]. Instead of increasing consumption of “traditional” plant-based food (e.g., pulses, vegetables, whole grains, etc.), an increasingly common strategy to facilitate and maintain the shift towards plant-based dietary patterns is to substitute animal products with plant-based analogues. These analogues are designed to mimic the sensory experiences of animal products, and thus these products could facilitate a transition towards plant-based dietary patterns since they could easily substitute animal foods [16,17]. Food companies often market their plant-based food analogues highlighting their environmental and nutritional benefits, for example, with “vegetarian/vegan/plant-based” claims and protein claims on packaging. As a result, thanks to a possible halo effect surrounding plant-based analogues (PBA), consumers often perceive plant-based alternatives as nutritionally adequate and/or environmentally friendly [18,19]. However, it is unclear whether these plant-based alternatives to animal food could provide the same or even increased benefits, also considering that there is limited information about the impact of substituting animal foods with plant-based products on intakes of nutrients and on health [20,21]. Among PBA, the market for dairy alternatives, especially of plant-based drinks (PBD), has expanded rapidly in Europe [22]. In Italy, for example, from 2005/2006 to 2017, the number of milk consumers decreased from 75% to 61% [23], while sales of PBD in food retail increased from 184 million euros in 2019 to 220 million euros in 2021 [24]. At the same time, there is a reduction in cow’s milk consumption falling below the amount recommended by current national dietary guidelines [25]. Lactose intolerance, milk protein allergies, cultural reasons, diet selection (e.g., veganism and flexitarian diets), concerns about animal welfare and climate change are the main reasons behind the substitution of cow’s milk (CM) with PBD [26]. Although PBD are used by consumers as alternatives to CM, several studies have observed that many PBD are not comparable or equivalent to CM in terms of nutritional composition [26,27,28,29,30]. Additionally, the nutritional composition of PBD can vary greatly among different types and even within the same PBD category depending on raw ingredients, transformation, and fortification processes. PBD can be fortified, e.g., with calcium, to provide the same amount of this mineral as in CM [28]. Consequently, due to the variability of the PBD, it is crucial to consider the products available in the local market when assessing the potential impact on nutrient intake following their use as a substitute for CM [31]. In this regard, there is a lack of data indicating the nutritional effect of this substitution [21,32] and the potential implications on human health.

Based on these premises, the aim of the present work was to assess the effect of substituting the consumption of CM with PBD currently sold in Italy on the potential nutrient intake associated with two different healthy and sustainable dietary patterns—the first in line with the ELHRD adapted to Italian food habits (EAT-IT) and the second in line with the Italian Dietary Guidelines (IDG) [11]—in order to detect any potential unintended consequence on nutrient intake deriving from the replacement of CM with PBD. Specifically, this study mainly focused on calcium content since cow’s milk plays a crucial role in supplying this micronutrient [26]. This analysis will provide further insights by examining the nutritional implications of a growing dietary trend, providing valuable data that can orient public health recommendations and steer consumer choices, making it a significant resource for researchers, policymakers, and health-conscious individuals.

## 2. Materials and Methods

### 2.1. PBD Selection and Data Extraction

Nutritional data of a total of 368 PBD currently available on the Italian market were collected from the FLIP database [27]. Data were further updated until July 2023. In addition to the mandatory nutrition information outlined in the Council Regulation (EU) 1169/2011, when available, information regarding fiber content (g/100 g) and other nutrients reported on the PBD label (i.e., calcium (mg/100 mL) and vitamin B12 (μg/100 mL) contents) was collected. Nutritional information on semi-skimmed milk was obtained from the IEO database “Food Composition Database for Epidemiological Studies in Italy” (Banca Dati di Composizione degli Alimenti per Studi Epidemiologici in Italia—BDA) [33].

After collecting the different data, a dataset was created by categorizing products into six groups according to the descriptive name: (i) soy drinks; (ii) almond drinks; (iii) oat drinks; (iv) rice drinks; (v) blend drinks (≥2 plant-based ingredients); (vi) other single ingredients (e.g., coconut, spelt, walnut drinks). Then, each category was further divided in two sub-groups: fortified with calcium (Ca-fortified) and not fortified with calcium (nCa-fortified).

At the end of the process, 12 categories that included the main Ca- and nCa-fortified PBD were obtained.

### 2.2. Selection of Representative Diets and Substitution of CM with PBD

For the present study, two dietary patterns (EAT-IT and IDG) developed on an energy target of 2500 kcal/day as previously reported were considered [11]. Specifically, two 7-day dietary plans for each pattern were formulated to assess the impact resulting from replacing CM with PBD on nutrient intake. In both models, CM consumption was planned only for breakfast, as already described by Tucci et al. [11]. However, for our purpose, some modifications to the original dietary patterns were adopted. These modifications included (1) the selection of semi-skimmed CM due to the major similarities between this product and PBD with a serving of 330 mL/day being one of the isocaloric alternatives to 250 mL of whole milk [7,11] and (2) the inclusion of 3 servings of 125 mL of CM instead of 2 servings of CM and 1 of yogurt as recommended by the Italian Dietary Guidelines [34].

After calculating the average nutritional value based on information declared on the food labels of the 12 aforementioned categories, a substitution of semi-skimmed CM was performed with an equivalent quantity in grams of each PBD category in both the CM IDG and CM EAT-IT dietary patterns. Specifically, in the IDG dietary pattern, the total CM intake was 375 mL/day (i.e., 3 servings of 125 mL each), while in the EAT-IT dietary pattern, it was 330 mL/day. Consequently, 12 different diets were obtained:

- With soy drinks (Ca- and not Ca-fortified)
- With almond drinks (Ca- and not Ca-fortified)
- With oat drinks (Ca- and not Ca-fortified)
- With rice drinks (Ca- and not Ca-fortified)
- With blend drinks (Ca- and not Ca-fortified)
- With other single ingredients drinks (Ca- and not Ca-fortified)

The elaboration of the diets and the estimation of their nutritional composition were conducted using MetaDieta Software (MètaDieta professional 4.1.1 METEDA Srl–Roma, Italy). The comparison of potential nutrient intake between the CM EAT-IT and the CM IDG, also considering substitution with Ca-fortified and nCa-fortified PBD, was performed using only those nutrients with data indicated on the label.

### 2.3. Evaluation of the Nutritional Adequacy of the IDG and EAT-IT Dietary Patterns with Both CM and PBD

After replacing CM with each PBD category within both the IDG and EAT-IT dietary patterns, the energy, macro- and micronutrients provided by all the diets were compared with the Italian Dietary Reference Values (DRVs) developed by the Italian Society of Human Nutrition (SINU) to assess whether these re-elaborated dietary patterns met the DRVs for the general Italian population [35]. Nutrient intake was expressed as mean ± standard deviation (SD), with SD representing the variability when the single items present in the Italian marker are included in the dietary models.

## 3. Results

### 3.1. Type and Nutritional Composition of PBD Currently Sold in Italy

A total of 368 PBD were collected from retailers present in the Italian market. Of these, products were mostly oat-based (22 out of the 81 were fortified with Ca) and soy-based (35 out of the 81 were fortified with Ca), followed by rice-based (12 out of the 59 were Ca-fortified) and blend-based PBD (15 out of the 57 were Ca-fortified).

In addition, 49 almond drinks (of which 11 were Ca-fortified) and 41 PBD belonged to “other single ingredients” (i.e., produced by single ingredients less represented such as coconut, spelt, quinoa) were collected.

### 3.2. Comparison of Nutritional Adequacy of the EAT-IT and IDG Dietary Patterns with CM or PBD

After replacing CM with PBD in both dietary patterns, the impact on the intake of nutrients indicated on the labels of PBD was assessed (Table 1, Table 2, Table 3, Table 4, Table 5, Table 6, Table 7 and Table 8). Concerning micronutrients, voluntarily expressed on the food pack, in addition to calcium (Ca), only those fortified in many PBD were reported: vit. B2 (*n* = 51), vit. B12 (*n* = 74), and vit. D (*n* = 85).

### 3.3. Energy and Macronutrients Provided by the EAT-IT and IDG Dietary Patterns with CM or PBD

Except for the Ca-fortified rice EAT-IT and IDG, all the other Ca-fortified PBD decreased the estimated energy intake compared to the CM EAT-IT (2501 kcal) and CM IDG (2427 kcal) dietary patterns, respectively (Table 1 and Table 2). In the case of not-fortified products, substituting CM with nCa-fortified oat, nCa-fortified rice, and an nCa-fortified blend resulted in a higher energy intake in both the EAT-IT and IDG dietary patterns. The remaining nCa-fortified PBD patterns showed a reduction in energy intake compared to both CM dietary patterns (Table 3 and Table 4).

In terms of macronutrients, replacing CM with all PBD resulted in a decrease in SFA intake. Additionally, it led to a reduced intake of protein with all PBD, except for the nCa-fortified soy drink, for which the substitution had no impact on the protein level. Contrariwise, the intake of fiber increased in both dietary patterns. The variation in the remaining macronutrients (lipid, carbohydrates, and sugars) following the substitution of CM with PBD depended on the type of PBD considered, as shown in Table 1, Table 2, Table 3 and Table 4.

Nutrient intakes calculated following the EAT-IT and IDG dietary patterns with CM and with PBD were then compared with the Italian DRVs [35] to verify their ability to meet the nutritional requirements. Overall, the dietary patterns analyzed could ensure an adequate nutrient intake able to satisfy the nutritional requirements with few exceptions as described below.

Regarding the EAT-IT based dietary patterns, those including the Ca-fortified single ingredients and both the Ca-fortified and nCa-fortified almond, provided comparable amounts of lipids almost equal to the higher level of the reference intake (RI) of 20–35% (i.e., 35.2%, 35.2%, and 35.7%, respectively). Furthermore, in both the Ca-fortified and nCa-fortified soy EAT-IT dietary patterns, carbohydrates provided 44.8% and 44.5% of energy, slightly below the RI (45–60% energy). In the CM EAT-IT and Ca-fortified rice EAT-IT dietary patterns, the energy from sugars corresponded to the SDT of 15%, while in the nCa-fortified rice EAT-IT dietary pattern, it was just slightly above (15.2%). Finally, the level of fiber intake was slightly higher than the RI (12.6–16.7 g/1000 kcal) both in the CM EAT-IT and all PBD EAT-IT dietary patterns. Considering the IDG dietary patterns, in both the CM and all PBD IDG ones, energy from sugars was slightly above the SDT (15%). Finally, the amount of fiber provided by IDG patterns in which CM was substituted with Ca-fortified oat, Ca-fortified single-ingredient, and Ca-fortified blend drinks, and nCa-fortified soy drinks, and Ca-fortified and nCa-fortified almond drinks was slightly above the RI. The substitution of CM with Ca-fortified soy, Ca-fortified and nCa-fortified rice, nCa-fortified oat, nCa-fortified single-ingredient, and nCa-fortified blend drinks resulted in dietary patterns with a fiber amount within the RI.

### 3.4. Micronutrients Provided by EAT-IT and IDG Dietary Patterns with CM or PBD

Regarding the micronutrients intake, the levels of vit. B2 and vit. B12 decreased when CM was replaced with all types of PBD. The amount of Ca, as well as of vit. D, depended on the type of PBD (Table 5, Table 6, Table 7 and Table 8).

Specifically, substituting CM with Ca-fortified soy and Ca-fortified rice drinks provided a slightly greater amount of Ca in both EAT-IT dietary patterns. In contrast, the replacement with Ca-fortified almond, Ca-fortified oat, and Ca-fortified single-ingredient drinks resulted in an equal Ca content of 839 mg and 1068 mg in the EAT-IT and in IDG dietary patterns, respectively. The substitution of CM with a Ca-fortified blend instead led to a reduced Ca intake within the EAT-IT and IDG dietary patterns, respectively (Table 5 and Table 6). Conversely, when CM was replaced with all nCa-fortified PBD in both EAT-IT and IDG patterns, the resulting dietary patterns provided a lower amount of Ca (Table 7 and Table 8).

After substituting CM with all Ca-fortified PBD, the levels of vit. D were higher than those in the CM EAT-IT and CM IDG dietary patterns (Table 5 and Table 6). When this substitution was carried out within the EAT-IT dietary pattern with nCa-fortified almond, nCa-fortified blend, nCa-fortified rice, and nCa-fortified a single-ingredient drinks, the amount of vit. D provided was equal to that of the CM EAT-IT plan (1.8 µg). Conversely, when CM was replaced with nCa-fortified soy and nCa-fortified oat drinks in the EAT-IT dietary pattern, the intake of vit. D increased (Table 7). Compared to the CM IDG dietary pattern, the substitution of CM with all nCa-fortified PBD did not affect the vit. D amount, except for nCa-fortified oat drinks, which led to a higher intake of vit. D (Table 8).

Subsequently, the micronutrients intake provided by these dietary patterns, both with CM and with all PBD, were compared with the Italian DRVs [35] to assess their ability to meet the nutritional requirements.

From this evaluation, it emerged that in all the diets, both with CM and with all PBD, the intake of vit. D was lower than the average requirement indicated by the LARN (10 µg) (Table 5, Table 6, Table 7 and Table 8).

In terms of Ca levels, both the EAT-IT and IDG dietary patterns in which CM was replaced with nCa-fortified PBD showed insufficient Ca levels to satisfy both the average requirement (AR) (800 mg) and population reference intake (PRI) (1000 mg) (Table 7 and Table 8). All Ca-fortified PBD EAT-IT patterns provided a Ca amount adequate to satisfy the AR but not the PRI (Table 5), whereas all Ca-fortified IDG dietary patterns led to an amount of Ca sufficient to meet both the AR and PRI (Table 6). Additionally, even the CM EAT-IT dietary pattern failed to provide an adequate amount of Ca when compared to the PRI, although it met the AR. On other hand, Ca provided by CM IDG dietary pattern was found to be adequate, even when compared to the PRI. In the IDG dietary patterns, both with CM and all PBD, adequate levels of vit. B2 were observed.

However, in the EAT-IT dietary patterns, this was not consistently observed. Specifically, exceptions were identified: all nCa-fortified dietary patterns were found to be inadequate for both the PRI for males (1.6 mg) and the PRI for females (1.3 mg), as well as for the AR for males (1.3 mg), except for the nCa-fortified oat plan which provided 1.3 mg of vit. B2. Furthermore, Ca-fortified rice, Ca-fortified single-ingredient, and Ca-fortified blend patterns also provided inadequate levels of vit. B2 when compared to the PRI for males (Table 5).

Finally, in all the dietary patterns considered, the amount of vit. B12 was found to be adequate.

## 4. Discussion

The continuous expansion of the market for plant-based substitutes to animal products, such as CM, reflects the growing number of consumers now inclined to decrease or stop the consumption of animal products, driven by concerns for the environment, health, and ethical issues [16]. However, despite this growing trend, the impact of substituting animal products with their plant-based counterparts on nutrient intake, nutritional status, and health is largely unknown due to the lack of longitudinal studies and randomized controlled trials [21,32].

In the present theoretical study, we tried to assess the role of CM within two dietary patterns (EAT-IT and IDG) and the effects of this substitution with PBD sold in Italy on nutrient intake. The choice of the patterns was performed in order to define the theoretical impact of PBD in the context of sustainable healthy diets considering today’s national dietary recommendations (IDG) and a pattern with the lowest environmental impact (EAT-IT). Following the substitution of CM with all PBD, macronutrient intake underwent only slight modifications, often not compromising the ability to meet the reference intake (RI) or the suggested dietary target (SDT). Overall, this substitution resulted in a reduced intake of protein and SFA, accompanied by an increased intake of dietary fiber. These findings are consistent with results from other studies [36,37]. Despite being higher, it is worth noting that values of SFA in CM dietary patterns did not exceed the related SDT (<10% En). However, the reduction in SFA occurring from the replacement of CM with PBD could also be viewed as a health advantage. A recent review suggested that the substitution of CM with soy drink may have a potential protective role in the modulation of the lipid profile. In total, five of the eight studies analyzing the effect of substituting CM with soy drink on the lipid profile included in this work showed that the consumption of soy drinks resulted in a reduction in low-density lipoprotein cholesterol (LDL-C), especially in hypercholesterolemic and overweight/obese subjects [32]. Nevertheless, there has been a recent debate about limiting intakes of SFA to less than 10% of total daily energy intake, as recommended by most dietary guidelines [38,39,40].

Traditionally, it has been suggested that SFA consumption increases the risk of CVD through the rise of LDL [38], despite recent research having shown that this relationship is not as clear. Furthermore, different SFAs seem to have different biological effects, which are further influenced by the food matrix and the type of dietary pattern [41,42]. In this context, Astrup et al., showed that the consumption of whole-fat dairy, rich in SFA and characterized by a complex matrix, was not associated with an increased risk of CVD [41]; similarly, when investigating the existing evidence on dairy foods consumption and health outcomes, Godos and coworkers found that a higher intake of total dairy foods was associated with a decreased risk of CVD [43]. Another change resulting from the substitution of CM with all PBD, except for soy drinks, within both the EAT-IT and IDG dietary patterns, was a reduction in protein intake, although still following the recommendations. This is in line with the Dietary Guidelines for Americans, which include only SD within the dairy group when properly fortified with vit. D, vit. A, and Ca. However, this study analyzes only the quantity of nutrients, but differences may also relate to their quality [31]. For instance, it has been shown that PBD exhibit a reduced digestible indispensable amino acid score compared to CM [30]; in fact, compared to animal proteins, plant proteins typically exhibit lower quality, characterized by a less favorable amino acid profile and reduced bioavailability [44]. Therefore, such differences should be considered when assessing the implications of substituting CM with PBD.

Regarding the micronutrient intake, the replacement of CM with PBD in both dietary patterns mainly led to a decreased intake of micronutrients, and some of them failed to reach the recommendations. Due to the important contribution of CM as a dietary source of Ca, the substitution of CM with all nCa-fortified PBD within both the EAT-IT and IDG dietary patterns led to low levels of Ca intake compared to the AR for adults (i.e., 800 mg/day).

Levels of Ca intake following the substitution of CM with Ca-fortified PBD were always adequate to meet the AR; however, Ca-fortified PBD EAT-IT dietary patterns were not able to satisfy the PRI. This result seems in line with those of Tucci et al. [11], who found that the main critical issue of the EAT-IT dietary pattern was a low Ca content not meeting the average requirements for adults (i.e., 800 mg/day). The low Ca intake was attributed to a low inclusion of CM and cheese in EAT-IT [11]. The important role of CM in contributing to adequate levels of Ca is also highlighted by the fact that, in comparison to the original EAT-IT dietary pattern, which included 250 mL of whole milk [11], in the CM EAT-IT plan we used an isocaloric quantity of semi-skimmed milk (330 mL), and this modification increased Ca levels sufficiently to meet the AR.

Even if levels of Ca were found to be nutritionally adequate when the substitution was made with Ca-fortified PBD, it is realistic to assume that consumers may not be aware of the importance of choosing Ca-fortified PBD, and, consequently, this substitution is unlikely to ensure the fulfilment of Ca needs. Therefore, it is important for consumers to be informed and to read food labeling to make informed food choices [45].

It is interesting to note that the majority of Ca-fortified PBD patterns (Ca-fortified almond, Ca-fortified oat, and Ca-fortified single-ingredient dietary patterns) provided the same level of Ca as the CM EAT-IT and CM IDG patterns, while the substitution of CM with Ca-fortified soy and Ca-fortified rice drinks led to higher levels of Ca compared to CM dietary patterns; only Ca-fortified blend dietary patterns resulted in reduced levels of Ca. This observation aligns with the results of several studies that demonstrate that beverages are often fortified to levels equal to or higher than the Ca level of CM [31,46,47]. In fact, Craig and colleagues demonstrated that the substitution of CM with a fortified plant-based drink does not results in a change in calcium intake within the “planetary diet” [37]. However, when comparing CM with Ca-fortified PBD, it is important to consider not only the amount of Ca but also the type of fortification used and the effect of the food matrix [48,49]. In fact, while milk and dairy products have a high content of bioavailable calcium [50], the bioavailability of Ca is not always comparable between CM and Ca-fortified PBD since different types of fortification can have different bioavailability. In detail, tri-calcium phosphate has only 75% the absorption of CM, while calcium carbonate, which nowadays is more commonly used, has a rate of absorption equal to the Ca provided by CM [31,51,52,53]. In addition, the presence of compounds such as myo-inositol phosphates, phytate, and oxalate in PBD may interfere with the absorption of Ca as well as other minerals and vitamins, reducing their absorption [54,55]. Furthermore, the sedimentation of Ca fortified in PBD reduces the amount of Ca provided by PBD since a certain amount of it remains in the settled residue [51]. Therefore, although the Ca content in Ca-fortified PBD matches that of CM, it remains uncertain if plant-based milk alternatives contain equivalent levels of bioavailable Ca compared with CM [31]. Further studies should analyze the types of Ca used in fortified PBD to understand their ability to be absorbed and, perhaps, to encourage food industries to use only highly absorbable forms of Ca in fortified PBD.

Also, the levels of vit. B2 and vit. B12 provided by all the PBD EAT-IT and all the PBD IDG dietary patterns were lower than those provided by the same dietary patterns with CM. The reduction in vit. B12 levels due to the substitution of CM with PBD is supported by two other studies [36,37]. However, their findings on vit. B2 levels differ. Craig et al. found that the substitution of CM with fortified PBD leads to increased vit. B2 intake [37]; in contrast, Clegg and colleagues demonstrated that vit. B2 levels varied depending on the type of PBD: they increased with coconut drink but decreased with PBD based on legumes, grains, nuts and seeds, and mixed ingredients [36].

Regarding nutritional adequacy, our study found that vit. B2 was inadequate in some PBD dietary patterns, while vit. B12 intake met the nutritional requirements. In detail, the EAT-IT diet in which CM was substituted with all nCa-fortified PBD, except for the nCa-fortified oat drink, provided a lower amount of vit. B2 compared to the AR for males as defined by LARN (i.e., 1.2 mg).

The level of vit. D provided was mainly dependent on the presence of Ca fortification in PBD. We observed that most Ca-fortified PBD were also fortified with vit. D, which is generally used in higher amounts compared to that naturally provided by CM. However, it is noteworthy that in this study the CM used was not fortified with vit. D even though the practice of fortification is becoming more common. Therefore, levels of vit. D in Ca-fortified PBD dietary patterns were higher than those found in CM dietary patterns. This is consistent with the results of Craig et al., which found that substituting CM with fortified PBD almost doubles vit. D values [37]. Compared to the Italian recommendations, vit. D levels were found to be lower than the AR (10 µg) in all dietary patterns, both those with CM and those with PBD. Although the major source of vit. D is endogenous synthesis, with food sources playing a relatively minor role in the total contribution, for some people (e.g., older subjects) the reduced intake of vit. D could represent a critical issue [56].

The results of this study should be analyzed considering some limitations. Firstly, the current work is limited to the highly dynamic PBD market. Although we collected quite a large set of PBD sold in Italy, other references may be present on the Italian market in stores not included in the present evaluation, thus not precisely reflecting the current market. Secondly, the comparison was limited to nutrients present on the food label. This limitation is significant because nutrient-based dairy messages in Food-Based Dietary Guidelines are related to 12 dairy-derived nutrients (protein; vitamins A, B2, B12, D; Ca, choline, iodine, phosphorus, potassium, selenium, and zinc), of which six are recognized as some of the most under-consumed nutrients globally (vitamins A and D, iodine, zinc, calcium, and potassium) [57]. For example, a recent dietary modeling study highlighted that replacing milk consumption with PBD sold in UK has the potential to reduce the iodine intake of the UK population, putting certain vulnerable population groups at risk [58]. However, other important components present in CM should be taken into consideration to better elucidate the impact of its substitution with PBD. In this regard, CM is an important source of α-lactalbumin, fat globule membranes, oligosaccharides, monounsaturated and polyunsaturated fatty acids (*n*-6 and *n*-3), gangliosides, and phospholipids, with some of them having an important impact on human health. For instance, milk oligosaccharides and whey proteins have been shown to positively modulate gut microbiota, thus contributing to the restoration of healthy microbiota and several associated health conditions [59]. Conversely, the presence of potentially harmful compounds, such as microRNA, deserves future investigation [60].

Therefore, limiting our analysis to nutrients listed on the food label could reduce the possibility of evaluating the real effect of this substitution on the nutrient intake of consumers and their nutritional and health status. Additionally, we assessed only the quantity of nutrients without considering their different bioavailability. Therefore, considering what was mentioned above, some of the reduced levels observed could even be worsened. This is particularly concerning considering that, in this study, two already optimal dietary patterns (i.e., based on IDG or EAT-IT) were utilized. In fact, the effect of such a substitution considering the actual consumption of the Italian population or sub-groups could not be necessarily the same. This aspect deserves further investigation in future experimental studies specifically aimed at exploring the real in vivo effect of substituting CM with PBD. However, to our knowledge, this study represents the first assessment of the impact of the substitution for CM with many PBD sold in Italy within healthy sustainable dietary patterns, providing important evidence about possible unintended nutritional inadequacies deriving from these substitutions.

## 5. Conclusions

In conclusion, our findings suggest that, due to the significant contribution of CM in providing nutrients, especially Ca, its replacement with PBD may negatively affect nutrient intake in sustainable dietary patterns, with an effect largely depending on the nutritional profile of single PBD on the market. Thus, the use of PBD as a substitute for CM should be carefully considered by taking into consideration the nutritional characteristics of these products, the presence of important components in CM but not in PBD, as well as the characteristics of the target group of the population. Consumers should be properly informed about the differences between CM and PBD, even within the same category of PBD, empowering them to make informed choices when substituting CM. Moreover, it is essential to encourage food companies to develop PBD fortified with appropriate levels of bioavailable Ca and other nutrients. Also given the growing popularity of PBD among consumers, further studies are needed to fully explore the impact of these substitutions and to identify possible unintended consequences of this replacement, taking into consideration the content of fundamental components in CM that are lacking in PBD.

## Figures and Tables

**Table 1:** Comparison between the energy and macronutrients provided by EAT-IT dietary pattern with CM and by the diets in which CM was substituted with Ca-fortified PBD. Daily reference intake of energy and nutrients for the Italian population (LARN) is reported for the assessment of nutritional adequacy of the analyzed diets [35].

	CM EAT-IT Plan	Ca Soy Plan ^1^	Ca Almond Plan ^1^	Ca Oat Plan ^1^	Ca Rice Plan ^1^	Ca Single-Ingredient Plan ^1^	Ca Blend Plan ^1^	LARN (Adults)
Energy (kcal)	2501	2497 ± 33	2434 ± 49	2503 ± 32	2536 ± 41	2435 ± 37	2464 ± 40	
Protein (g)	101.9 ^a^	101.2 ± 1.6 ^a^	92.2 ± 1.3 ^a^	92.3 ± 1.2 ^a^	91.5 ± 2.7 ^a^	91.0 ± 0.8 ^a^	92.4 ± 2.0 ^a^	AR 0.71 g/kg × die (PRI 0.9 g/kg × die 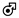 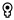 )
Energy protein/total energy (%)	16.3	16.2 ± 0.2	15.2 ± 0.2	14.8 ± 0.2	14.4 ± 0.4	14.9 ± 0.1	15.0 ± 0.3	12–18% En
Lipids (g)	95.4	97.1 ± 1.7	95.2 ± 3.2	95.9 ± 2.3	94.7 ± 2.3	95.3 ± 2.0	94.8 ± 2.1	
Energy lipids/total energy (%)	34.3	35.0 ± 0.4	35.2 ± 0.7 ^a^	34.5 ± 0.5	33.6 ± 0.5	35.2 ± 0.5 ^a^	34.6 ± 0.5	RI 20–35% En
Saturated fat (g)	16.5	14.7 ± 0.3	14.0 ± 0.3	14.3 ± 0.2	14.8 ± 2.3	15.6 ± 1.2	15.1 ± 1.8	
Energy SFA/total energy (%)	5.9	5.3 ± 0.1	5.2 ± 0.1	5.1 ± 0.1	5.3 ± 0.8	5.8 ± 0.4	5.5 ± 0.6	SDT < 10% En
Carbohydrates (g)	284.6	279.5 ± 7.0	277.2 ± 10.6	292.0 ± 5.0	304.8 ± 9.8	278.4 ± 5.8	285.6 ± 11.2	
Energy carbohydrates/total energy (%)	45.5	44.8 ± 0.6 ^a^	45.5 ± 0.9	46.7 ± 0.4	48.1 ± 0.8	45.7 ± 0.5	46.4 ± 1.0	RI 45–60% En
Sugars (g)	93.7	86.5 ± 6.9	84.3 ± 9.4	89.0 ± 6.2	95.1 ± 6.7	84.3 ± 4.5	85.7 ± 5.3	
Energy sugars/total energy (%)	15.0 ^a^	13.9 ± 0.9	13.9 ± 1.3	14.2 ± 0.9	15.0 ± 0.9 ^a^	13.8 ± 0.6	13.9 ± 0.7	SDT < 15% En
Dietary fiber (g)	44.1	45.4 ± 1.3	44.7 ± 0.5	45.8 ± 1.7	44.4 ± 0.4	44.7 ± 0.6	45.0 ± 0.9	
Total fiber/1000 kcal (g)	17.6 ^a^	18.2 ± 0.5 ^a^	18.4 ± 0.2 ^a^	18.3 ± 0.7 ^a^	17.5 ± 0.1 ^a^	18.4 ± 0.3 ^a^	18.3 ± 0.4 ^a^	RI 12.6–16.7 g/1000 kcal

Legend: AR: average requirement; Ca: calcium-fortified; CM: cow’s milk; EAT-IT: dietary pattern in line with the EAT-Lancet Commission reference diet adapted to Italian food habits; En: energy; LARN: reference intake of nutrients and energy for the Italian population; PBD: plant-based drinks; PRI: population reference intake; RI: reference intake; SDT: standard dietary target; SFA: saturated fatty acids; ^1^ plan: EAT-IT dietary pattern in which CM was replaced with Ca-fortified PBD; ^a^: the level of intake for the respective nutrient was above or below the nutritional requirements, depending on the nutrient; 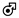: equivalent to 63 g/die for males; 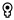: equivalent to 54 g/die for females.

**Table 2:** Comparison between the energy and macronutrients provided by IDG dietary pattern with CM and by these diets in which CM was substituted with Ca-fortified PBD. Daily reference intake of energy and nutrients for the Italian population (LARN) is reported for the assessment of nutritional adequacy of the analyzed diets [35].

	CM IDG Plan	Ca Soy Plan ^1^	Ca Almond Plan ^1^	Ca Oat Plan ^1^	Ca Rice Plan ^1^	Ca Single-Ingredient Plan ^1^	Ca Blend Plan ^1^	LARN (Adults)
Energy (kcal)	2427	2423 ± 38	2351 ± 56	2430 ± 37	2467 ± 46	2352 ± 39	2386 ± 46	
Protein (g)	97.2	96.5 ± 1.9	86.3 ± 1.4	86.3 ± 1.3	85.4 ± 3.0	84.8 ± 0.9	86.4 ± 2.2	AR 0.71 g/kg × die (PRI 0.9 g/kg × die 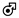 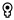 )
Energy protein/total energy (%)	16.0	15.9 ± 0.3	14.7 ± 0.2	14.2 ± 0.2	13.8 ± 0.4	14.4 ± 0.1	14.5 ± 0.3	12–18% En
Lipids (g)	75.7	77.6 ± 1.9	75.4 ± 3.6	76.2 ± 2.6	74.8 ± 2.6	75.5 ± 2.2	75.0 ± 2.4	
Energy lipids/total energy (%)	28.1	28.8 ± 0.5	28.9 ± 0.9	28.2 ± 0.7	27.3 ± 0.7	27.9 ± 0.6	28.3 ± 0.6	RI 20–35% En
Saturated fat (g)	18.8	16.7 ± 0.4	15.9 ± 0.3	16.3 ± 0.3	16.8 ± 2.6	17.8 ± 1.4	17.1 ± 2.1	
Energy SFA/total energy (%)	7.0	6.2 ± 0.1	6.1 ± 0.1	6.0 ± 0.1	6.1 ± 0.9	6.6 ± 0.5	6.5 ± 0.7	SDT < 10% En
Carbohydrates (g)	319.1	313.3 ± 8.0	310.7 ± 12.1	327.6 ± 5.7	342.0 ± 11.1	312.0 ± 6.5	320.3 ± 12.7	
Energy carbohydrates/total energy (%)	52.6	51.7 ± 0.6	52.9 ± 0.9	53.9 ± 0.4	55.5 ± 0.8	53.1 ± 0.5	53.7 ± 1.0	RI 45–60% En
Sugars (g)	113.4	105.3 ± 7.9	102.7 ± 10.7	108.2 ± 7.1	115.0 ± 7.6	102.8 ± 5.1	104.4 ± 6.0	
Energy sugars/total energy (%)	18.7 ^a^	17.4 ± 1.1 ^a^	17.5 ± 1.5 ^a^	17.8 ± 1.0 ^a^	18.6 ± 1.0 ^a^	17.5 ± 0.7 ^a^	17.5 ± 0.8 ^a^	SDT < 15% En
Dietary fiber (g)	39.1	40.5 ± 1.5	39.7 ± 0.5	40.9 ± 2.0	39.3 ± 0.4	39.7 ± 0.7	40.0 ± 1.1	
Total fiber/1000 kcal (g)	16.1	16.7 ± 0.6	16.9 ± 0.2 ^a^	16.8 ± 0.8 ^a^	15.9 ± 0.2	16.9 ± 0.3 ^a^	16.8 ± 0.5 ^a^	RI 12.6–16.7 g/1000 kcal

Legend: AR: average requirement; Ca: calcium-fortified; CM: cow’s milk; En: energy; IDG: dietary pattern in line with the Italian Dietary Guidelines; LARN: Reference Intake of Nutrients and Energy for the Italian Population; PBD: plant-based drinks; PRI: population reference intake; RI: reference intake; SDT: standard dietary target; SFA: saturated fatty acids; ^1^ plan: IDG dietary pattern in which CM was replaced with Ca-fortified PBD; ^a^: the level of intake for the respective nutrient was above or below the nutritional requirements, depending on the nutrient; 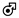: equivalent to 63 g/die for males; 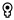: equivalent to 54 g/die for females.

**Table 3:** Comparison between the energy and macronutrients provided by EAT-IT dietary pattern with CM and by the diets in which CM was substituted with nCa-fortified PBD. Daily reference intake of energy and nutrients for the Italian population (LARN) is reported for the assessment of nutritional adequacy of the analyzed diets [35].

	CM EAT-IT Plan	nCa Soy Plan ^1^	nCa Almond Plan ^1^	nCa Oat Plan ^1^	nCa Rice Plan ^1^	nCa Single- Ingredients Plan ^1^	nCa Blend Plan ^1^	LARN (Adults)
Energy (kcal)	2501	2494 ± 35	2467 ± 57	2510 ± 33	2536 ± 30	2497 ± 49	2542 ± 48	
Protein (g)	101.9 ^a^	101.9 ± 1.6 ^a^	93.2 ± 1.7 ^a^	92.6 ± 1.0 ^a^	91.8 ± 1.7 ^a^	92.5 ± 1.7 ^a^	93.1 ± 2.7 ^a^	AR 0.71 g/kg × die (PRI 0.9 g/kg × die 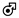 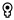 )
Energy protein/total energy (%)	16.3	16.3 ± 0.2	15.1 ± 0.2	14.8 ± 0.1	14.5 ± 0.2	14.8 ± 0.2	14.6 ± 0.4	12–18% En
Lipids (g)	95.4	97.0 ± 1.1	97.8 ± 3.5	95.1 ± 1.6	93.9 ± 1.1	97.0 ± 6.6	96.7 ± 2.5	
Energy lipids/total energy (%)	34.3	35.0 ± 0.3	35.7 ± 0.8 ^a^	34.1 ± 0.4	33.3 ± 0.3	35.0 ± 1.4	34.2 ± 0.6	RI 20–35% En
Saturated fat (g)	16.5	14.9 ± 0.5	14.3 ± 0.4	14.4 ± 0.4	14.3 ± 0.6	15.6 ± 2.5	15.0 ± 1.1	
Energy SFA/total energy (%)	5.9	5.4 ± 0.2	5.2 ± 0.1	5.2 ± 0.1	5.1 ± 0.2	5.6 ± 0.8	5.3 ± 0.4	SDT < 10% En
Carbohydrates (g)	284.6	277.7 ± 7.1	278.6 ± 10	295.8 ± 5.7	306.7 ± 6.7	291.2 ± 14.1	300.8 ± 10.7	SDT < 300 mg
Energy carbohydrates/total energy (%)	45.5	44.5 ± 0.6 ^a^	45.2 ± 0.9	47.1 ± 0.5	48.4 ± 0.5	46.6 ± 1.2	47.3 ± 0.9	RI 45–60% En
Sugars (g)	93.7	84.3 ± 7.5	84.4 ± 8.2	93.0 ± 6.2	96.4 ± 7.4	89.3 ± 8.6	94.1 ± 6.1	
Energy sugars/total energy (%)	15.0 ^a^	13.5 ± 1.0	13.7 ± 1.1	14.8 ± 0.8	15.2 ± 1.0 ^a^	14.3 ± 1.2	14.8 ± 0.8	SDT < 15% En
Dietary fiber (g)	44.1	46.2 ± 2.3	45.1 ± 1.0	45.6 ± 1.4	44.8 ± 1.0	45.0 ± 1.0	45.4 ± 1.5	
Total fiber/1000 kcal (g)	17.6 ^a^	18.5 ± 0.9 ^a^	18.3 ± 0.4 ^a^	18.2 ± 0.6 ^a^	17.7 ± 0.4 ^a^	18.0 ± 0.4 ^a^	17.9 ± 0.6 ^a^	RI 12.6–16.7 g/1000 kcal

Legend: AR: average requirement; CM: cow’s milk; EAT-IT: dietary pattern in line with the EAT-Lancet Commission reference diet adapted to Italian food habits; En: energy; LARN: reference intake of nutrients and energy for the Italian population; nCa: not calcium-fortified; PBD: plant-based drinks; PRI: population reference intake; RI: reference intake; SDT: standard dietary target; SFA: saturated fatty acids; ^1^ plan: IDG dietary pattern in which CM was replaced with nCa-fortified PBD; ^a^: the level of intake for the respective nutrient was above or below the nutritional requirements, depending on the nutrient; 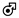: equivalent to 63 g/die for males; 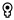: equivalent to 54 g/die for females.

**Table 4:** Comparison between the energy and macronutrients provided by IDG dietary pattern with CM and by the diets in which CM was substituted with nCa-fortified PBD. Daily reference intake of energy and nutrients for the Italian population (LARN) is reported for the assessment of nutritional adequacy of the analyzed diets [35].

	CM IDG Plan	nCa Soy Plan ^1^	nCa Almond Plan ^1^	nCa Oat Plan ^1^	nCa Rice Plan ^1^	nCa Single- Ingredients Plan ^1^	nCa Blends Plan ^1^	LARN (Adults)
Energy (kcal)	2427	2420 ± 39	2389 ± 65	2438 ± 38	2467 ± 34	2423 ± 55	2474 ± 54	
Protein (g)	97.2 ^a^	97.2 ± 1.8 ^a^	87.4 ± 1.9 ^a^	86.7 ± 1.2 ^a^	85.8 ± 1.9 ^a^	86.6 ± 2.0 ^a^	87.3 ± 3.1 ^a^	AR 0.71 g/kg × die (PRI 0.9 g/kg × die 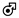 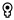 )
Energy protein/total energy (%)	16.0	16.1 ± 0.3	14.6 ± 0.3	14.2 ± 0.2	13.9 ± 0.3	14.3 ± 0.3	14.1 ± 0.4	12–18% En
Lipids (g)	75.7	77.5 ± 1.2	78.4 ± 4.0	75.3 ± 1.9	74.0 ± 1.3	77.5 ± 7.5	77.1 ± 2.8	
Energy lipids/total energy (%)	28.1	28.8 ± 0.3	29.5 ± 1.1	27.8 ± 0.5	27.0 ± 0.3	28.8 ± 1.8	28.0 ± 0.7	RI 20–35% En
Saturated fat (g)	18.8	16.9 ± 0.6	16.2 ± 0.4	16.4 ± 0.4	16.2 ± 0.7	17.8 ± 2.8	17.0 ± 1.3	
Energy SFA/total energy (%)	7.0	6.3 ± 0.2	6.1 ± 0.1	6.1 ± 0.1	5.9 ± 0.2	6.6 ± 1.0	6.2 ± 0.4	SDT < 10% En
Carbohydrates (g)	319.1	311.2 ± 8.1	312.2 ± 11.4	331.8 ± 6.4	344.2 ± 7.6	326.6 ± 16.0	337.5 ± 12.2	
Energy carbohydrates/total energy (%)	52.6	51.4 ± 0.6	52.3 ± 0.9	54.4 ± 0.5	55.8 ± 0.5	53.9 ± 1.2	54.6 ± 0.9	RI 45–60% En
Sugars (g)	113.4	102.8 ± 8.5	102.9 ± 9.4	112.6 ± 7.0	116.6 ± 8.4	108.5 ± 9.7	113.9 ± 6.9	
Energy sugars/total energy (%)	18.7 ^a^	17.0 ± 1.1 ^a^	17.2 ± 1.3 ^a^	18.5 ± 0.9 ^a^	18.9 ± 1.1 ^a^	17.9 ± 1.3 ^a^	18.4 ± 0.9 ^a^	SDT < 15% En
Dietary fiber (g)	39.1	41.4 ± 2.6	40.1 ± 1.1	40.7 ± 1.6	39.8 ± 1.2	40.0 ± 1.1	40.5 ± 1.7	
Total fiber/1000 kcal (g)	16.1	17.1 ± 1.1 ^a^	16.8 ± 0.5 ^a^	16.7 ± 0.7	16.1 ± 0.5	16.5 ± 0.4	16.4 ± 0.7	RI 12.6–16.7 g/1000 kcal

Legend: AR: average requirement; CM: cow’s milk; En: energy; IDG: dietary pattern in line with the Italian Dietary Guidelines; LARN: reference intake of nutrients and energy for the Italian population; nCa: not calcium-fortified; PBD: plant-based drinks; PRI: population reference intake; RI: reference intake; SDT: standard dietary target; SFA: saturated fatty acids; ^1^ plan: IDG dietary pattern in which CM was replaced with nCa-fortified PBD; ^a^: the level of intake for the respective nutrient was above or below the nutritional requirements, depending on the nutrient; 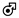: equivalent to 63 g/die for males; 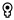: equivalent to 54 g/die for females.

**Table 5:** Comparison between the micronutrients provided by EAT-IT dietary pattern with CM and by the diets in which CM was substituted with Ca-fortified PBD. Daily reference intake of energy and nutrients for the Italian population (LARN) is reported for the assessment of nutritional adequacy of the analyzed diets [35].

	CM EAT-IT Plan	Ca Soy Plan ^1^	Ca Almond Plan ^1^	Ca Oat Plan ^1^	Ca Rice Plan ^1^	Ca Single-Ingredient Plan ^1^	Ca Blend Plan ^1^	LARN	
								AR	PRI
Vit. D (µg) (cholecalciferol, ergocalciferol)	1.8 ^a^	4.0 ± 1.3 ^a^	4.2 ± 1.6 ^a^	4.4 ± 1.7 ^a^	3.5 ± 1.9 ^a^	4.6 ± 1.3 ^a^	3.6 ± 1.3 ^a^	10 µg	15 µg
Vit. B2 (mg) (riboflavin)	1.8	1.7 ± 0.3	1.7 ± 0.3	1.6 ± 0.3	1.3 ± 0.2 ^b^	1.5 ± 0.3 ^b^	1.3 ± 0.2 ^b^	Males 1.3 mg (Females 1.1 mg)	Males 1.6 mg (Females 1.3 mg)
Vit. B12 (µg) (cyanocobalamin)	4.0	3.5 ± 0.6	3.6 ± 0.6	3.5 ± 0.6	3.2 ± 0.6	3.8 ± 0.2	3.2 ± 0.6	2 µg	2.4 µg
								AR	PRI
Calcium (mg)	839.1 ^c^	853.1 ± 39.1 ^d^	839.1 ± 0.0 ^d^	839.1 ± 0.0 ^d^	850.1 ± 38.1 ^d^	839.1 ± 0.0 ^d^	828.5 ± 34.3 ^d^	800 mg	1000 mg

Legend: AR: average requirement; Ca: calcium-fortified; CM: cow’s milk; EAT-IT: dietary pattern in line with the EAT-Lancet Commission reference diet adapted to Italian food habits; PBD: plant-based drinks; PRI: population reference intake; ^1^ plan: EAT-IT dietary pattern in which CM was replaced with Ca PBD; ^a^: the level of intake for the respective nutrient was inadequate to satisfy the nutritional requirements; ^b^: the level of intake for the respective nutrient was insufficient to meet PRI for females; ^c^: the level of intake for the respective nutrient was insufficient to meet PRI for males; ^d^: the level of intake for the respective nutrient was insufficient to meet PRI.

**Table 6:** Comparison between the micronutrients provided by IDG dietary pattern with CM and by the diets in which CM was substituted with Ca PBD. Daily reference intake of energy and nutrients for the Italian population (LARN) is reported for the assessment of nutritional adequacy of the analyzed diets [35].

	CM IDG Plan	Ca Soy Plan ^1^	Ca Almond Plan ^1^	Ca Oat Plan ^1^	Ca Rice Plan ^1^	Ca Single- Ingredients Plan ^1^	Ca Blend Plan ^1^	LARN	
								AR	PRI
Vit. D (µg) (cholecalciferol, ergocalciferol)	2.2 ^a^	4.7 ± 1.5 ^a^	4.9 ± 1.8 ^a^	5.2 ± 1.9 ^a^	4.0 ± 2.2 ^a^	5.4 ± 1.5 ^a^	4.2 ± 1.4 ^a^	10 µg	15 µg
Vit. B2 (mg) (riboflavin)	2.3	2.2 ± 0.4	2.2 ± 0.4	2.1 ± 0.4	1.7 ± 0.2	1.9 ± 0.4	1.7 ± 0.2	Males 1.3 mg (Females 1.1 mg)	Males 1.6 mg (Females 1.3 mg)
Vit. B12 (µg) (cyanocobalamin)	4.8	4.3 ± 0.7	4.3 ± 0.7	4.3 ± 0.6	3.9 ± 0.7	4.6 ± 0.3	3.9 ± 0.7	2 µg	2.4 µg
								AR	PRI
Calcium (mg)	1067.8	1083.8 ± 44.4	1067.8 ± 0.0	1067.8 ± 0.0	1080.3 ± 43.3	1067.8 ± 0.0	1055.8 ± 39.0	800 mg	1000 mg

Legend: AR: average requirement; Ca: calcium-fortified; CM: cow’s milk; IDG: dietary pattern in line with the Italian Dietary Guidelines; PBD: plant-based drinks; plan: IDG dietary pattern in which CM was replaced with Ca PBD; PRI: population reference intake; ^1^ plan: IDG dietary pattern in which CM was replaced with Ca-fortified PBD; ^a^: the level of intake for the respective nutrient was inadequate to satisfy the nutritional requirements.

**Table 7:** Comparison between the micronutrients provided by EAT dietary pattern with CM and by the diets in which CM was substituted with nCa-fortified PBD. Daily reference intake of energy and nutrients for the Italian population (LARN) is reported for the assessment of nutritional adequacy of the analyzed diets [35].

	CM EAT-IT Plan	nCa Soy Plan ^1^	nCa Almond Plan ^1^	nCa Oat Plan ^1^	nCa Rice Plan ^1^	nCa Single- Ingredients Plan ^1^	nCa Blend Plan ^1^	LARN	
								AR	PRI
Vit. D (µg) (cholecalciferol, ergocalciferol)	1.8 ^a^	1.9 ± 0.4 ^a^	1.8 ± 0.0 ^a^	1.9 ± 0.5 ^a^	1.8 ± 0.0 ^a^	1.8 ± 0.0 ^a^	1.8 ± 0.0 ^a^	10 µg	15 µg
Vit. B2 (mg) (riboflavin)	1.8	1.2 ± 0.1 ^d^	1.2 ± 0.0 ^d^	1.3 ± 0.1 ^b^	1.2± 0.0 ^d^	1. 2 ± 0.0 ^d^	1.2 ± 0.0 ^d^	Males 1.3 mg (Females 1.1 mg)	Males 1.6 mg (Females 1.3 mg)
Vit. B12 (µg) (cyanocobalamin)	4.0	2.7 ± 0.2	2.7 ± 0.0	2.7 ± 0.2	2.7 ± 0.2	2. 7 ± 0.0	2.7 ± 0.0	2 µg	2.4 µg
								AR	PRI
Calcium (mg)	839.1 ^c^	443.1 ± 0.0 ^a^	443.1 ± 0.0 ^a^	443.1 ± 0.0 ^a^	443.1 ± 0.0 ^a^	443.1 ± 0.0 ^a^	443.1 ± 0.0 ^a^	800 mg	1000 mg

Legend: AR: average requirement; CM: cow’s milk; EAT-IT: dietary pattern in line with the EAT-Lancet Commission reference diet adapted to Italian food habits; nCa: not calcium-fortified; PBD: plant-based drinks; PRI: population reference intake; ^1^ plan: EAT-IT dietary pattern in which CM was replaced with nCa-fortified PBD; ^a^: the level of intake for the respective nutrient was inadequate to satisfy the nutritional requirements; ^b^: the level of intake for the respective nutrient was insufficient to meet PRI for males; ^c^: the level of intake for the respective nutrient was insufficient to meet PRI; ^d^: the level of intake for the respective nutrient was insufficient to meet AR for males and the PRI for both males and females.

**Table 8:** Comparison between the micronutrients provided by IDG dietary pattern with CM and by the diets in which CM was substituted with nCa-fortified PBD. Daily reference intake of energy and nutrients for the Italian population (LARN) is reported for the assessment of nutritional adequacy of the analyzed diets [35].

	CM IDG Plan	nCa Soy Plan ^1^	nCa Almond Plan ^1^	nCa Oat Plan ^1^	nCa Rice Plan ^1^	nCa Single- Ingredients Plan ^1^	nCa Blend Plan ^1^	LARN	
								AR	PRI
Vit. D (µg) (cholecalciferol, ergocalciferol)	2.2 ^a^	2.2 ± 0.4 ^a^	2.2 ± 0.0 ^a^	2.3 ± 0.5 ^a^	2.2 ± 0.0 ^a^	2.2 ± 0.0 ^a^	2.2 ± 0.0 ^a^	10 µg	15 µg
Vit. B2 (mg) (riboflavin)	2.3	1.7 ± 0.1	1.7 ± 0.0	1.7 ± 0.1	1.7 ± 0.0	1.7 ± 0.0	1.7 ± 0.0	Males 1.3 mg (Females 1.1 mg)	Males 1.6 mg (Females 1.3 mg)
Vit. B12 (µg) (cyanocobalamin)	4.8	3.3 ± 0.2	3.3 ± 0.0	3.3 ± 0.3	3.3 ± 0.2	3.3 ± 0.0	3.3 ± 0.0	2 µg	2.4 µg
								AR	PRI
Calcium (mg)	1067.8	617.8 ± 0.0 ^a^	617.8 ± 0.0 ^a^	617.8 ± 0.0 ^a^	617.8 ± 0.0 ^a^	617.8 ± 0.0 ^a^	617.8 ± 0.0 ^a^	800 mg	1000 mg

Legend: AR: average requirement; CM: cow’s milk; IDG: dietary pattern in line with the Italian Dietary Guidelines; nCa: not calcium-fortified; PBD: plant-based drinks; plan: IDG dietary pattern in which CM was replaced with nCa PBD; PRI: population reference intake; ^1^ plan: IDG dietary pattern in which CM was replaced with nCa-fortified PBD; ^a^: the level of intake for the respective nutrient was inadequate to satisfy the nutritional requirements.

## Data Availability

The original contributions presented in the study are included in the article, further inquiries can be directed to the corresponding author.
